# Dietary Management of Children With Super-Refractory Status Epilepticus: A Systematic Review and Experience in a Single UK Tertiary Centre

**DOI:** 10.3389/fneur.2021.643105

**Published:** 2021-03-12

**Authors:** Natasha E. Schoeler, Zoe Simpson, Runming Zhou, Suresh Pujar, Christin Eltze, J. H. Cross

**Affiliations:** ^1^Developmental Neurosciences Research and Teaching Department, University College London Great Ormond Street Institute of Child Health, London, United Kingdom; ^2^Department of Dietetics, Great Ormond Street Hospital for Children, London, United Kingdom; ^3^Department of Paediatric and Neurology, Great Ormond Street Hospital for Children, London, United Kingdom; ^4^Young Epilepsy, Lingfield, United Kingdom

**Keywords:** ketogenic, super-refractory status epilepticus, seizure, epilepsy, systematic review

## Abstract

Ketogenic diet therapies (KDT) are high-fat, low carbohydrate diets used as an effective treatment option for drug-resistant epilepsy. There is limited research on the efficacy of KDT for super-refractory status epilepticus (SRSE). We systematically review evidence for use of KDT in children with SRSE and present a single UK tertiary centre's experience. Thirty one articles were included, of which 24 were “medium” or “low” quality. One hundred and forty seven children with SRSE started KDT, of which 141 (96%) achieved ketosis. KDT was started mean 5.3 days (range 1–420) after status epilepticus (SE) started. SRSE resolved in 85/141 (60%) children after mean 6.3 days (range 0–19) post SE onset, but it is unclear whether further treatments were initiated post-KDT. 13/141 (9%) children died. Response to KDT was more likely when initiated earlier (*p* = 0.03) and in females (*p* = 0.01). Adverse side effects were reported in 48/141 (34%), mostly gastrointestinal; potentially serious adverse effects occurred in ≤4%. Eight children with SRSE, all diagnosed with febrile infection-related epilepsy syndrome, were treated with KDT at Great Ormond Street Hospital for Children. KDT was initiated enterally at mean day 13.6+/− 5.1 of admission. Seven of 8 (88%) children reported adverse side effects, which were potentially serious in 4/8 (50%), including metabolic acidosis, hypoglycaemia and raised amylase. SE ceased in 6/8 (75%) children after mean 25+/− 9.4 days post onset, but other treatments were often started concomitantly and all children started other treatments post-KDT. Two of 8 (25%) children died during admission and another died post-admission. Four of the remaining 5 children continue to have drug-resistant seizures, one of whom remains on KDT; seizure burden was unknown for one child. Our findings indicate that KDT is possible and safe in children with SRSE. Cessation of SRSE may occur in almost two-thirds of children initiated with KDT, but a causal effect is difficult to determine due to concomitant treatments, treatments started post-KDT and the variable length of time post-KDT onset when SRSE cessation occurs. Given that serious adverse side effects seem rare and response rates are (cautiously) favorable, KDT should be considered as an early treatment option in this group.

## Introduction

Ketogenic diet therapies (KDT) are a group of high-fat, low-carbohydrate diets used as a treatment option for drug-resistant epilepsy. Designed to mimic the effects of starvation on the body, fat is utilized as the principle energy source through production of ketones. Children with drug-resistant epilepsy treated with KDT have a relative risk of 3.16 of achieving seizure freedom, and a relative risk of 5.80 of achieving ≥50% seizure reduction compared to usual care (1).

Over the past decade, reports have increased of use of KDT in acute situations, in particular for super refractory status epilepticus (SRSE). SRSE, which is defined as status epilepticus (SE) that continues for at least 24 h after initiation of general anesthesia or that recurs after anesthesia reduction/withdrawal (2), is associated with high morbidity and a mortality rate of >30% (3). KDT is described as “particularly useful” for SRSE in international consensus guidelines, being consistently reported as “more beneficial (>70%) than the average 50% KDT response (defined as >50% seizure reduction)” (4). However, most reports of use of KDT in children with SRSE are from single centres, often only including individuals with single diagnoses, and no attempt to date has been made to collate published results.

We present a systematic review of the literature of use of KDT in children with SRSE, and describe our experience at a single UK tertiary centre with 8 children with SRSE who were started on KDT. We aimed to evaluate feasibility of initiation of KDT, safety and short- and long-term effectiveness, and to investigate characteristics associated with efficacy of KDT for SRSE in the literature.

## Methods

### Systematic Review

A systematic literature search was conducted in the electronic databases MEDLINE (PubMed) and Embase (Ovid) with the following keywords: ketogenic AND (“status epilepticus” OR “super refractory status epilepticus” OR SRSE OR “Febrile Infection-Related Epilepsy Syndrome” OR FIRES). Publications including children only (aged 19 years or under), written in English or Spanish were included; no date restrictions were set. Reference lists of publications, including reviews, were manually searched. The search was up-to-date as of 27th October 2020. This study was performed according to the Preferred Reporting Items for Systematic Reviews and Meta-Analyses (PRISMA) guidelines.

#### Eligibility Criteria

Controlled studies, uncontrolled cohort studies (prospective and retrospective), case series, case reports and letters/commentaries fulfilling the following criteria were included:

- Inclusion of at least one individual aged ≤18 years with SRSE (either described as “super-refractory status epilepticus” with the above definition, or evident that status epilepticus had been continuing for at least 24 h after initiation of general anesthesia or recurring with anesthesia reduction/discontinuation), in whom KDT was started;
- Clear clinical outcome after initiation of KDT, for example, resolution of SRSE or other seizure-related outcome.

Children with non-convulsive status and myoclonic status in non-progressive encephalopathy were not included.

#### Study Selection

Duplicate records were excluded. Titles and abstracts were screened for study eligibility, and full-text articles were reviewed by NS and RZ. Cases of disagreement were discussed until consensus was reached.

#### Quality Appraisal

Study quality was appraised using 12 criteria adapted from the Critical Appraisal Skills Programme (CASP) Cohort Study Checklist. This addresses 10 criteria via the use of 10 questions, plus 2 initial screening questions. Study quality was defined as follows: studies meeting 8–10 criteria were described as “high quality,” studies meeting 5–7 criteria were described as “medium quality,” and studies meeting <5 criteria were described as “low quality.” This approach has been used in previously published systematic reviews (5, 6). No studies were excluded based on CASP score, but rather the tool was used to identify methodological limitations of included studies.

#### Outcomes

The primary outcome was the number or proportion of children achieving resolution of SRSE or 100% seizure reduction post-initiation of KDT.

Secondary outcomes were:

- number or proportion of children deceased;
- SE duration prior to KDT start;
- number of treatments tried prior to KDT start;
- treatments tried post-KDT start, if mentioned;
- KDT ratio and level of ketosis achieved;
- seizure burden at latest follow-up;
- functional outcome at latest follow-up;
- adverse side effects from KDT.

#### Data Extraction

The following data (where available), were extracted for each study:

- study design;
- number of children with SRSE started on KDT;
- number of children with SRSE started on KDT but who did not achieve ketosis.
  For each participant, where available:
- gender;
- presence of previous epilepsy;
- age of onset of SE;
- etiology of SRSE;
- SE duration when KDT commenced;
- number of treatments (AEDs, anesthetic agents or other) received after onset of SE, prior to KDT start;
- time to reach ketosis;
- definition of ketosis;
- KDT ratio achieved;
- mode of feeding;
- KDT duration;
- adverse side effects;
- resolution of SE or other clinical outcome;
- (for those who achieved resolution of SE) days since SE onset when resolved;
- seizure burden at latest follow-up;
- functional status at latest follow-up.

#### Data Analysis

Descriptive analysis was conducted for primary and secondary outcomes; data were summarized as aggregate rates (with the number of participants who achieved some level of ketosis on KDT as the total), mean and ranges for numerical outcomes. Combined means were calculated from study means, weighted by the number of individuals in each study. A narrative syntheses was compiled of categorical outcomes, including reported side effects, seizure burden and functional outcome at latest follow-up.

Where per-patient data were available, age at SE onset, gender, SE duration prior to KDT initiation and diet ratio were compared for those who did and those who did not achieve resolution of SRSE or seizure freedom. Difference between means were compared using independent sample *t-*test and distribution in categorical variables were compared using Pearson's χ^2^ test.

### Case Note Review

A retrospective review of medical records was conducted of all children with SRSE started on KDT at Great Ormond Hospital for Children (GOSH) since 2010. The following data were extracted for each participant, where available: age at SE onset, anti-seizure drugs (ASDs) and other seizure treatments initiated prior to KDT, day of admission when KDT started, number of ASDs and other seizure treatments commenced during admission, KDT ratio at initiation and maximum ratio tolerated, time to obtain stable ketosis, time KDT followed, time post SE onset when SRSE resolved, length of admission, seizure burden, and functional status at latest follow-up.

## Results

### Systematic Review

**Figure 1:**
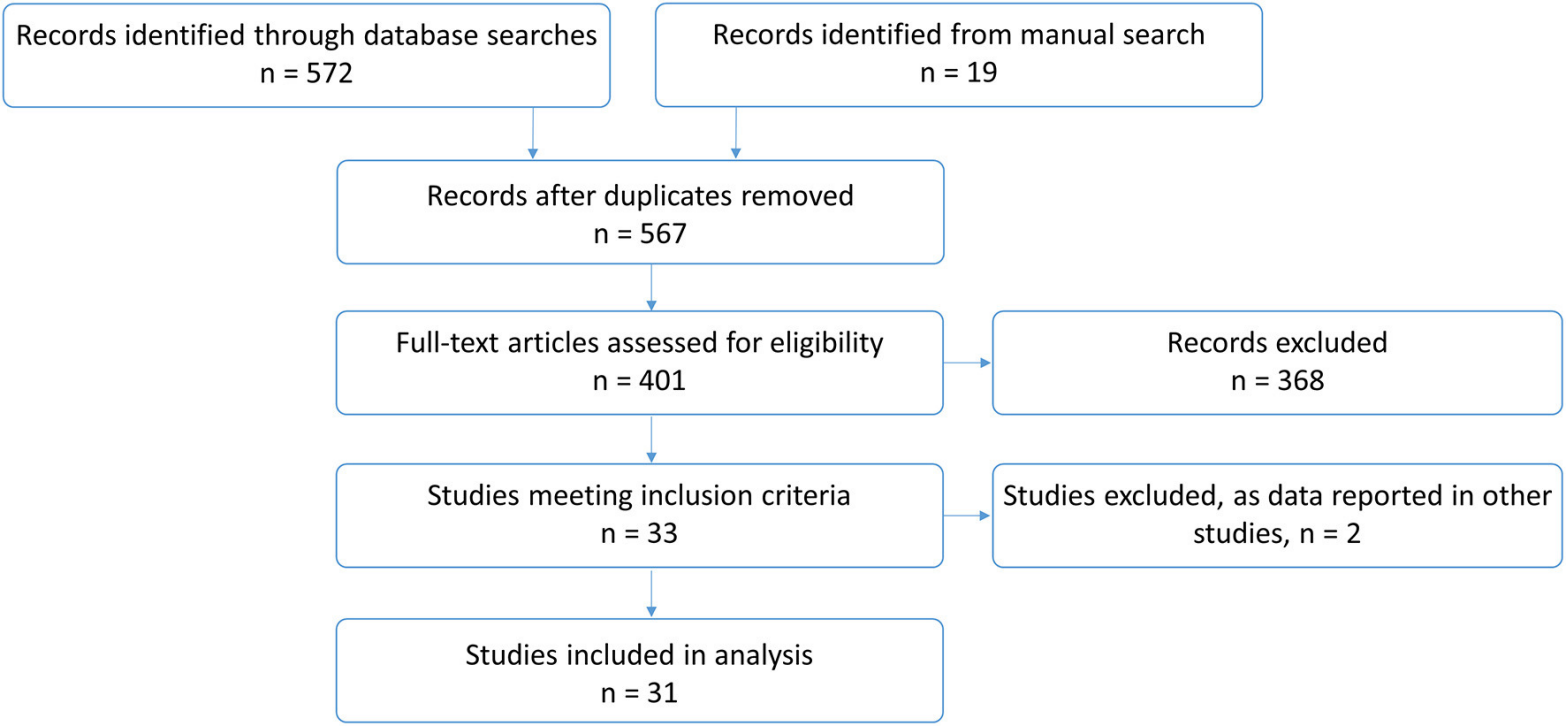
Flowchart of study selection.

In total, 591 publications were identified. After removing 24 duplicates, 401 studies were screened and assessed for eligibility. Two articles were excluded (7, 8) as participants were also reported in other included studies (9, 10) [and, for one article, response data were only given for one participant on KDT (7)]. Thirty one studies, which are listed in Table 1, were included in the final analysis (Figure 1).

**Table 1:** List of included studies from literature review, *n* = 31.

**First author and year of publication**	**Reference**
Chee, 2020	(11)
Chiu, 2020	(12)
Gupta, 2020	(13)
Wang, 2020	(14)
Dilena, 2019	(15)
Park, 2019	(9)
Peng, 2019	(16)
Lam, 2019	(17)
Arayakarnkul, 2019	(18)
Arya, 2018	(19)
Lee, 2018	(20)
Farias-Moeller, 2017	(21)
Appavu, 2016	(22)
Chiusolo, 2016	(23)
Appavu, 2016	(24)
Caraballo, 2016	(25)
Kenney-Jung, 2016	(26)
Fung, 2015	(27)
Lin, 2015	(28)
Moriyama, 2015	(29)
Cobo, 2015	(30)
Fung, 2014	(31)
O'Connor, 2014	(32)
Singh, 2014	(33)
Sort, 2013	(34)
Caraballo, 2013	(35)
Vaccarezza, 2012	(36)
Ismail, 2011	(37)
Kramer, 2011	(10)
Nabbout, 2010	(38)
Villeneuve, 2009	(39)

#### Overview of Results

Fourteen studies were retrospective, 1 was a prospective, observational cohort study, and 16 were described as case reports/studies. Supplementary Table 1 shows summary descriptive data for all included studies.

A total of 147 children with SRSE were started on KDT, of which 141 (96%) achieved ketosis.

60/114 (53%) children were male (information given in 25 studies); mean age at SE onset was 4.2 years (range 0.1–19, information given in 27 studies); 30/108 (28%) children had epilepsy prior to SRSE (information given in 26 studies) and the most common diagnosis/etiology of SRSE was febrile infection-related epilepsy syndrome (FIRES), reported in 72/126 (57%) children with known diagnosis/etiology.

Mean duration of SE prior to KDT start was 5.3 days (range 1–420, information given in 21 studies) and 3.2 treatments were tried prior to KDT start (range 1–11, information given in 16 studies). 98/113 (87%) children were fed KDT enterally, and 15/113 (13%) parenterally (information given in 19 studies), although two children fed parenterally then transitioned onto enteral feeding after a range of 3–5 days and one child started KDT for a second time, fed enterally.

A target 4:1 KDT ratio was reached by 74/89 (83%) children, 3–3.5:1 by 10/89 (11%), 2–2.75:1 by 3/89 (3%, of which one started KDT for a second time at 1:1), 5:1 by 1/89 (1%) and 6:1 by 1/89 (1%) (information given in 20 studies). Mean time to achieve ketosis was 3.4 days (range <1–20, information given in 14 studies), but the definition of ketosis were variable, for example, any presence of urinary ketones (“ketonuria”) or more specific definitions such as a “goal of >3 mmol/L serum beta-hydroxybutyrate” (information given in 12 studies).

KDT was followed for a mean of 4.5 months [range 0.1–15.8, information given in 17 studies, although diet duration was only reported for responders in one study (40)].

#### Study Quality

Seven studies met the criteria for “high quality,” although these were all observational and retrospective; 13 studies were “medium quality” and 11 studies were “low quality” (Supplementary Table 2).

#### Efficacy of KDT

85/141 (60%) children achieved resolution of SRSE (also described as seizure freedom) after initiation of KDT. One of these children had previously been started on KDT with no improvement. Another individual achieved resolution of SRSE but only after other therapies had been added post-KDT initiation (11), and so he was not counted as a responder. Other treatments started post-KDT were not detailed for other responders. Resolution of SRSE was achieved after mean 6.3 days (SD 4.4, range 0–19) post-KDT initiation. 4/141 (3%) children died in the acute stage of SRSE, one of whom had achieved prior resolution of SRSE. A further 9 children died post-discharge, bringing the total number of deaths at latest follow-up to 13/141 (9%).

At latest follow-up, 26/141 (18%) children were seizure-free, 66/141 (47%) had seizures, and the remainder had unknown seizure burden or had died. Of those who had seizures, 8/141 (6%) had rare/sporadic/occasional seizures, 17/1 (12%) had monthly seizures, 15/141 (11%) had weekly seizures, 17/141 (12%) had daily seizures and 9/141 (6%) had seizures of unspecified frequency.

At latest follow-up, 10/141 (7%) children were able to return to normal daily living or to “baseline,” 12/141 (9%) had mild mental retardation or cognitive impairment, 8/141 (6%) had moderate mental retardation/cognitive impairment/encephalopathy, 13/141 (9%) had severe mental retardation/cognitive impairment/encephalopathy, 6/141 (4%) were in a vegetative state or bedridden, and 11/141 (8%) had “other” functional difficulties that did not fit in the previous categories, including “behavioral problems” (*n* = 3), “mental retardation” (*n* = 2), hemiplegia (*n* = 2), “improved” alertness/eye contact/quality of life (*n* = 2), and unspecified severity of cognitive/learning impairment (*n* = 2). The remainder did not have functional outcome reported, or they had died.

#### Characteristics of KDT Efficacy

There was no significant difference in age at SE onset [*t* (101) = −0.69, *p* = 0.25, 1 tail[ or diet ratio [χ^2^ (4, *N* = 69) = 1.00, *p* = 0.75] between responders and non-responders (Table 2). Responders were more likely to be female [χ^2^ (1, *N* = 98) = 0.82, *p* = 0.01] and have KDT initiated earlier in the course of SRSE [*t* (96) = −1.90, *p* = 0.03, 1 tail] compared to non-responders (Table 2).

**Table 2:** Association of clinical and demographic variables with response to ketogenic diet therapies.

**Variable**	**Responders**	**Non-responders**	***P-*value**
Age at status epilepticus onset	*M =* 7.1, SD = 3.9, *n =* 67	*M =* 7.8, SD = 20.2, *n =* 38	0.25
Duration of status epilepticus prior to onset of ketogenic diet therapies	*M =* 16.2, SD = 15.2, *n =* 67	*M =* 38.7, SD = 79.5, *n =* 33	0.03^*^
Ketogenic diet ratio	5:1, *n =* 2 4:1–4.9:1, *n =* 41 3:1–3.9:1, *n =* 5 2:1–2.9:1, *n =* 1 1:1–1.9:1, *n =* 1	5:1, *n =* 0 4:1–4.9:1, *n =* 16 3:1–3.9:1, *n =* 3 2:1–2.9:1, *n =* 0 1:1–1.9:1, *n =* 0	0.75
Gender	Female, *n =* 36 Male, *n =* 27	Female, *n =* 11 Male, *n =* 24	0.01^*^

* *p <0.05*.

#### Adverse Effects

Adverse side effects were reported in 48/141 (34%) children. These were most commonly gastrointestinal [regurgitation, vomiting, constipation, diarrhea, abdominal distention, gastro-intestinal paresis, or gastroesophageal reflux, *n* = 13 (9%)], or hypertriglyceridemia or hyperlipaemia [*n* = 11 (8%)], although this occurred in the context of hemophagocytic lymphohistiocytosis for *n* = 1 and whilst taking valproate for *n* = 1. Other side effects were rare, including aspiration pneumonia (*n* = 6, 4%), other biochemical abnormalities (elevated liver enzymes, hypokalemia or hypoprotinaemia, *n* = 6, 4%), hypoglycaemia (*n* = 2, 1%), weight loss (*n* = 3, 2%), metabolic acidosis/ketoacidosis (*n* = 2, 1%) kidney stones/nephrolithiasis (*n* = 3, 2%, alongside topiramate for one of these two children), or pancreatitis (*n* = 1, 1%, whilst taking valproate), suspected sepsis (*n* = 1, 1%), arrhythmia (*n* = 1, 1%), haematuresis (*n* = 1, 1%), and increase breakthrough seizures (*n* = 1, 1%).

### Case Note Review

A total of 8 children were referred for initiation of ketogenic diet therapies (KDT) for super-refractory status epilepticus (SRSE) at our institution 2010–2020. Mean age on admission was 7.9 +/− 1.7 years (range 5.8–10.8) and 75% (6/8) were male (Table 3).

**Table 3:** Cohort clinical and demographic characteristics, *n* = 8.

**ID**	**Age at seizure onset (years)**	**Number of ASDs commenced during admission**	**Number of other seizure treatments commenced during admission**	**Number of treatments initiated prior to KDT**	**Day of admission when KDT started (days)**	**KDT ratio at initiation**	**Maximum KDT ratio tolerated with therapeutic ketosis**	**Time to obtain stable ketosis (days)**	**Length of KDT (days)**	**Resolution of SRSE (days)**	**Length of admission (days)**	**Seizure burden at latest follow-up**	**Functional status at latest follow-up**
1	10.8	10	3	9	13	4:1	2.5:1	3	15	n/a (deceased)	30	Deceased	Deceased
2	9.1	11	5	14	15	3:1	5:1	12	80	19	117	Monthly clusters of seizures	In mainstream school but with learning and behavioral difficulties
3	10.1	13	5	16	24	3.3:1	4:1	3	383 (ongoing)	28	166	Weekly seizures	Almost back to baseline, walks independently but gait unsteady
4	5.8	13	4	8	15	4:1	3:1	1	57	31	125	Unknown	Unknown
5	7.2	9	3	8	12	2:1	3:1	12	25	12	49	Deceased	Deceased
6	6.5	14	4	10	6	2.5:1	3:1	1	12	n/a (deceased)	19	Deceased	Deceased
7	6.9	11	3	9	13	2:1	4:1	3	18	Unknown	31	Daily seizures	Mixed dystonic/pyramidal motor disorder, not independently mobile, severe intellectual disability
8	6.7	8	3	Unknown	11	1:1	4:1	3	18	35	95	Daily seizures	4-limb motor disorder with spasticity and dystonia, wheelchair dependent, severe intellectual disability

*ASD, anti-seizure drug; KDT, ketogenic diet therapies; SRSE, super-refractory status epilepticus*.

Prior to admission, none of the children had epilepsy. All children presented with clinical characteristics of FIRES with rapid onset of SRSE following a febrile illness, requiring intubation and anesthesia. All children were diagnosed with FIRES at the time of treatment.

During admission, children were commenced on a mean of 11.1 +/− 2.0 (range 9–15) anti-seizure drugs (ASDs) and 3.9 +/− 0.83 (range 3–5) other treatments (Table 4). All children had immune modulating therapy, including intravenous immunoglobulin (*n* = 8), Anakinra (*n* = 4), or plasmapheresis (*n* = 5). Three children had surgical intervention either deep brain stimulation (*n* = 2) or vagus nerve stimulation (*n* = 1) started post-KDT. There was no pattern as to when treatments were introduced, and multiple treatments, including KDT, were often instigated together. Seven children had failed corticosteroids prior to the KDT being initiated. No children had KDT concurrently with corticosteroids.

**Table 4:** Number of children who received non-anti-seizure drug treatments during admission.

**Treatment**	**Number of children who received treatment, *n* (%)**
Methyl prednisolone	8 (100%)
Intravenous immunoglobulin	8 (100%)
Plasma exchange	5 (62.5%)
Anakinra	4 (50%)
Dexamethasone	2 (25%)
Deep brain stimulation	2 (25%)
Vagus nerve stimulation	1 (12.5%)
Therapeutic hypothermia	1 (12.5%)

Initiation of KDT occurred on 13.6 +/− 5.1 days (range 6–24) post-admission. The mean number of treatments for SRSE prior to KDT was 11.1 +/− 3.4. All children were commenced on the classical ketogenic diet (CKD) via nasogastric tube. The CKD was initiated at a ratio between 1:1 and 4:1. The CKD ratio tolerated by children with production of therapeutic ketosis (2–6 mmol/L) without significant adverse side effects ranged between 2.5:1 and 5:1. The mean CKD ratio tolerated was 3.4:1 (range 2.5:1–5:1). Two children had medium chain triglyceride (MCT) included as part of their KDT on initiation and one had MCT commenced during their admission. It took a mean of 4.75 +/− 4.6 days (range 1–12) to gain stable therapeutic ketosis.

Two children died during admission, one at 19 days and one 30 days post-admission. Of the six children who survived, clinical and/or electrographic (on EEG) resolution of SE occurred at mean day 25 +/− 9.4 post onset (range 12–35, time unknown for one child). Four of these 6 children (67%) remained on KDT at the time of SE resolution (unknown for *n* = 1). The mean length of hospital stay for survivors was 97.2 +/− 50.3 days (range 31–166). Post SE EEGs remained abnormal for all children, and all continued to have a high seizure burden.

Of 8 children, 7 (88%) exhibited adverse side effects that could potentially be related to initiation of KDT (Table 5). Four children had a potentially serious adverse side effect (metabolic acidosis, hypoglycaemia or raised amylase, lipase +/− lactate dehydrogenase). All but one child discontinued KDT during admission. Mean duration of KDT for the children who have stopped diet (7) was 32.3 +/− 26 days (range 12–80). KDT was discontinued in 3 children due to uncertain efficacy in controlling seizures, one child due to non-compliance with KDT post-discharge from intensive care, one child was unable to maintain therapeutic ketosis, and 2 children died.

**Table 5:** Adverse side effects potentially associated with ketogenic diet therapies.

**Adverse side effect**	**Number of children with reported adverse effect, *n* (%)**
Loose stool	5 (62.5%)
Metabolic acidosis	4 (50%)
Hyperketosis (≥6 mmol/l)	3 (37.5%)
Hypertriglycerideamia	3 (37.5%)
Raised amylase and lipase	1 (12.5%)
Raised amylase, lipase, and lactate dehydrogenase	1 (12.5%)
Weight loss	1 (12.5%)
Hypoglycaemia (≤3 mmol/l)	1 (12.5%)

Longer-term follow up (mean 4.8 +/− 3.1 years, range 0.5–8.5) information was available for 5 children. One child died 7 years after acute presentation with FIRES due to an unrelated condition. The remaining 4 patients continue to have seizures. Two patients have daily seizures, one has weekly seizures and the other experiences a patterns of monthly seizure clusters. All children remained on ASDs, with intellectual and motor impairments impairment ranging from moderate to severe. One child remains on KDT to date.

## Discussion

Our systematic literature review and single-centre experience showed that KDT is feasible and safe to implement in children with SRSE, as long as they are monitored for potential adverse side effects. KDT can be an effective treatment option in the acute stage of SRSE although published literature is mostly of medium/low quality and, as in our centre's experience, KDT is not always effective. Long-term outcomes in terms of seizure burden and functional status for children with SRSE remain poor.

Initiation of KDT is feasible in intensive care units, administered either enterally or parenterally, with 96% of children in the literature and all 8 children in our clinical cohort achieving ketosis. KDT may have been successfully implemented in all children in our clinical cohort, as corticosteroids were not given concomitantly, although KDT was started relatively late in the clinical evolution compared to cases in the literature. Details regarding concomitant medications were not given in published studies for those in whom ketosis could not be achieved. Data was also insufficient from the literature review to warrant conclusions to be drawn regarding optimal levels of ketosis. Even when achieved, a consistent level of ketosis may not always be easy to maintain in a critical care setting, due to concomitant carbohydrate-containing medications, corticosteroids, barbiturates, anesthesia or insulin resistance.

KDT was generally safe to use in this critically-ill cohort, with the majority of adverse side effects being gastrointestinal or an unfavorable lipid profile, which were usually transient or resolved with medical management. Unfavorable lipid profiles have been shown to normalize 6–12 months after starting KDT in non-acute situations, and to return to baseline following diet discontinuation (41, 42). Not all serious adverse side effects reported in the literature were necessarily attributable to KDT, such as sepsis and aspiration pneumonia. Serious adverse side effects that are known to occur with non-acute use of KDT for epilepsy, including renal stones, acidosis and hypoglycaemia, seem to occur at a similar (and rare) rate as in acute situations, with an incidence of 1–3% (43), and often in conjunction with ASDs that also increase side effect risk.

Resolution of SRSE or seizure cessation rates post-KDT initiation in almost two-thirds of children reported in the literature is certainly promising. However, response is difficult to consistently attribute to KDT due to the variable length of time post-diet initiation that SRSE resolved or seizures ceased, together with lack of information regarding treatments started post-KDT start. Resolution of SRSE cannot be attributed to KDT in any of the 8 children in our single centre experience, as SRSE did not cease until after initiation of other treatments after KDT had been started. This may have been due to the fact that KDT was started relatively late in the course of SRSE in most of the children because of the time taken to determine the effect of treatment with various anti-seizure medications and, in particular, corticosteroids. Other reasons for the apparent discrepancy in response rates between the literature and our centre's experience include potential publication bias against unsuccessful cases, variable definitions of response reported in the literature (clinical observable seizures or electrographic seizures), and the natural course of SRSE, with remission of SE independent from interventions.

Our analysis of per-patient data from the literature revealed that earlier initiation of KDT and female gender were associated with increased likelihood of resolution of SRSE post-KDT initiation. Greater response rates with early initiation may be due to the natural course of SRSE, with the less refractory cases being over-represented. Differing response rates with gender may be associated with particular SRSE etiologies, or may be a chance finding, particularly as the number of cases with KDT response and gender data was limited. Barriers to early implementation of KDT in acute settings may include a lack of familiarity amongst neurologists regarding KDT as a treatment option, the need for propofol infusion, contraindicated with use of KDT (44), time taken to obtain results of metabolic investigations excluding the possibility of fatty-acid beta-oxidation disorders and, in some centres, a lack of trained, specialist dietitians. Despite cautiously optimistic efficacy rates, the majority of survivors continued to have drug-resistant seizures in the longer term. Functional outcomes also appear poor, with <10% returning to “normal” or baseline, but relevant data are lacking in the literature. Poor seizure-related and functional outcome may be related to “late” cessation of SE [earlier cessation is associated with better outcomes (45)] and are likely linked to SRSE etiology.

There are several limitations of this review. No randomized controlled trials were identified—studies were all observational, mostly retrospective, cohort studies or case reports/series and predominantly of medium or low quality. No studies compared KDT to no change in treatment although, considering the high mortality and morbidity of SRSE, clinicians are faced with the question of starting KDT or trying another ASD or alternative seizure treatment and high-quality evidence comparing these two treatment options may be more appropriate. Details regarding timings of other treatments initiated and discontinued, both before, during and after KDT, were limited, which may have impacted diet efficacy rates. Furthermore, few publications provided details regarding long-term functional outcome. More children with SRSE have been treated with KDT and are published in the literature, but without age-specific diet efficacy data and so have not been included in this review, such as Worden et al. (46). Alongside this data collection bias, publication bias against negative results must also be considered. The single-centre case note review is limited by the nature of retrospective data collection, in particular missing data regarding initiation and discontinuation dates of ASDs and other treatments.

In conclusion, evidence for efficacy of KDT for the treatment of SRSE is limited, with low quality data suggestive of benefits. The timing of KDT induction earlier in the course of SRSE may well be crucial for its efficacy. Prospective data (including detailed information on concomitant treatments) using a standardized agreed protocol are required to confirm this. Serious adverse side effects are rare, but close monitoring is needed.

## Data Availability Statement

The raw data supporting the conclusions of this article will be made available by the authors, without undue reservation.

## Author Contributions

NS wrote the manuscript and was the first reviewer for the systematic review. RZ acted as second reviewer for the systematic review. ZS collated the clinical data and helped to write the manuscript. SP, CE, and JC critically reviewed the manuscript. All authors contributed to the article and approved the submitted version.

## Conflict of Interest

NS is supported for a research post by Vitaflo (International) Ltd, and has received honoraria from Vitaflo (International) Ltd and Nutricia, outside the submitted work. JC has acted as an investigator for studies with GW Pharma, Zogenix, Vitaflo, and Marinius. She has been a speaker and on advisory boards for GW Pharma, Zogenix, and Nutricia; all remuneration has been paid to her department. The remaining authors declare that the research was conducted in the absence of any commercial or financial relationships that could be construed as a potential conflict of interest.
